# Cardiovascular adverse events associated with immune checkpoint inhibitors: a meta-analysis

**DOI:** 10.3389/fimmu.2024.1394123

**Published:** 2024-06-24

**Authors:** Xi Li, Dan Li

**Affiliations:** Cancer Center, The First Hospital of Jilin University, Changchun, China

**Keywords:** ICIS, cardiovascular, adverse events, anti-cancer, pericarditis, myocarditis

## Abstract

**Objective:**

To evaluate the cardiovascular safety of anticancer drug immune checkpoint inhibitors (ICIs) used in patients with malignant tumors.

**Methods:**

Four clinical research databases that have been completed since their establishment were searched, and the odds ratios and 95% confidence intervals of each indicator were statistically calculated.

**Results:**

62 randomized controlled trial and controlled trials were included. In single drug treatment ICIs group, the overall risk of cardio cerebral Vascular disease at all levels was higher than that in the placebo/chemotherapy group. Especially in all grades of Myocarditis and above grade 3 compared with normal controls, except for pericardial lesions, other indicators have no obvious side effects.

**Conclusion:**

Single drug use of an anti-tumor ICIs may increase cardiovascular side effects risk in cancer patients, so we need to strengthen monitoring, identification and management, and timely intervention to manage ICI induced adverse events.

## 1 Introduction

During tumors developing, certain genes in the tumor tissue will gradually undergo changes, resulting in the production of many new antigens. The natural and acquired immune system can recognize and inhibit tumor growth. But by selecting non immune tumor cells or suppressing immune responses, cancer cells can avoid immune monitoring. The research on stimulating the anti tumor immune response of the body has a history of decades. However, due to various factors, the initial efforts did not have much effect. Among them, multiple signaling pathways such as CTLA-4, PD-1, and PD-L1 significantly reduced the cytotoxicity of T cells. These signaling pathways are significant in maintaining the normal function of the body’s immune system and preventing the occurrence of autoimmune diseases. Tumors escape the specific immune response of T lymphocytes through these signaling pathways (1). Therefore, activating these checkpoints is considered an effective method to explain the escape of cancer cells from the immune system. ICI is an emerging anticancer drug that activates the body’s immune system, prevents its immune escape from tumors, and prompts the body to kill them. Although ICI has a good anti-cancer effect, it may cause inflammatory reaction in the body, and trigger “inflammatory storm”, resulting in multiple organ damage, which is a toxic side effect related to immunity. The human body has a strong tolerance to most of the toxic and side effects (such as rash, Thyroiditis, etc.) (2). However, some sudden immune toxic side effects, such as heart disease (HD), have received high attention due to their severe clinical manifestations and high mortality rate. Among them, ICI Myocarditis is the most common, with an incidence of more than 0.05% and a mortality rate of more than 30%. So far, literature reports and long-term follow-up studies on Myocarditis related to ICI at home and abroad are not adequate (3). The evaluation of cardiac function before and during the implementation of ICI has gradually become a research hotspot, especially in the early stages. In recent years, due to the progress of HD research, more knowledge about ICI preventing and treating combined with Myocarditis has existed (4). However, in the follow-up search, scholars have different views on the CAE caused by ICIs, and some literatures have different standards when extracting data. This study will evaluate the risk of CAE caused by ICIs through Randomized controlled trial, and try to explore the relationship between ICIs and their overall treatment strategies.

## 2 Materials and methods

### 2.1 Literature search

This study was developed and implemented based on the principles of systematic evaluation and meta-analysis reporting. The researchers independently searched all Randomized controlled trial in Embase, PubMed and Co hrane library databases since the establishment of database. The selected search terms cotain ICIs, PD-1, PD-L1, CTLA-4, tisliezumab, cemiplimab, atezolizumab, duralumab, tremelimumab, pembrolizumab, ipilimumamb, nivolimumab, cardiac toxicity, mycocardis.

### 2.2 Selection criteria and research choices

Inclusion criteria: Randomized controlled trial of CAE after receiving ICIs treatment. The included Randomized controlled trial can be classified into: studies comparing single drug ICIs and placebo/chemotherapy, single drug ICIs combination chemotherapy and simple chemotherapy, and dual drug ICIs and single drug ICIs/chemotherapy. To conduct a more rigorous analysis of the cardiovascular side effects of ICIs on their users, this trial only includes reported, well documented, extractable, randomized, and controlled trials. The researchers conducted separate reviews of the titles and abstracts of the identified papers, and provided a complete evaluation of meaningful papers. If there are any different opinions, they will be discussed with the co authors. Exclusion criteria: This study excluded repeated published experiments. Articles in the form of case reports, conference abstracts, or non treatment related CAE papers were not included.

### 2.3 Quality evaluation

The study independently evaluated the bias risk of each included article using Cochrane guidelines, and calculated the included article’s bias risk based on software RevMan5.4.1. They include randomly generated and assigned hidden biases (selection bias), subject and blind methods (execution bias), selective result reporting (reporting bias), and other biases.

### 2.4 Data extraction

After searching the literature and reading all parts of the article (such as the title, abstract and full text), the cardiovascular diseases related to Immunosuppressive drug treatment referred to in the article are extracted and summarized. The extracted data include: National Clinical Trials (NCT), article title, research author, cancer type, treatment time, trial stage and category, trial combination control group (CG) scheme, and frequency of ICIs related cardiovascular diseases.

### 2.5 Results

HD events (heart failure, cardiac arrest, myocardial ischemia/infarction, myocarditis, pericardial disease, supraventricular arrhythmia, valvular disease) and vascular disease events (hypertension, Hypotension, Vasculitis, thrombosis, etc.) caused by ICIs were comprehensively evaluated. For precise evaluation, all clinical trials were divided into several parts. Based on different drug treatments, corresponding treatment plans were evaluated. They include single drug ICIs vs placebo, single drug ICIs vs non ICIs, single drug ICIs+non ICIs vs non ICIs, dual drug ICIs vs single drug ICIs, and dual drug ICIs vs non ICIs.

### 2.6 Data analysis

Data are statistically analyzed using Review Manager version 5.4.1, and patients were classified and grouped based on drug treatment plans. The Mantel Haenszel method calculates odds ratio (OR) and corresponding 95% confidence interval. Q statistics and I2 values can be applied to evaluation, and *P*<0.05 or I2 greater than 75% were used as statistically significant differences. For individuals with significant differences, a random influence pattern was used, whereas a fixed influence pattern was used. *P* is a double tailed type with a significance of 0.05 set. When discovering samples with significant differences, the samples with significant differences are excluded, or these samples are individually excluded, and then a new statistical analysis is conducted to evaluate their sensitivity. SPSS 24.0 was selected for comparative analysis of two treatment methods.

## 3 Result analysis

### 3.1 Adverse events evaluation results for all levels

According to the selected query entries, there are a total of 4423 papers in four databases and various clinical research institutions. After reading the title, abstract, full text, and supplementary materials, a total of 62 papers were selected. The total number of cases N is 37823, cases T’s number in experimental group (EG) is 19894, and cases C’s number in control group(CG) is 17929. CAE related to ICIs mainly includes cardiac function and vascular function. According to the classification of commonly used adverse event (AE) evaluation criteria, cardiac AEs include valve disease, arrhythmia, cardiomyopathy, pericardial disease, myocardial infarction, and cardiac dysfunction. Under these diseases, cardiac AE is further classified into various HD. Hypertension, Hypotension, lymphatic leakage, lymphedema, Lymphatic vessel, peripheral ischemia, phlebitis, superficial thrombophlebitis, thromboembolism and Vasculitis are common vascular AEs. The purpose of this experiment is to evaluate the role of ICIs in the occurrence of adverse cardiovascular reactions in tumor patients. **Table 1** provides a summary of the efficacy evaluation of each level of AE. In the single drug ICIs group, there were significant differences in overall adverse reactions and vascular adverse reactions compared to CG. Although there was varying degrees of heterogeneity among studies, the sensitivity of overall cardiac AE was the same. In addition, the data also suggest that the common CAE related to ICIs is mainly myocarditis and pericardial diseases.

**Table 1:** Summary of AE evaluations at all levels.

AE	Single drug ICI			Dual drug ICI		
	EG	CG	P	EG	CG	P
CAE	218(1.15)	781(5.13)	<0.05	8(0.46)	14(0.58)	0.36
heart failure	34(0.18)	24(0.16)	0.43	1(0.03)	1(0.04)	0.87
Cardiac arrest	17(0.09)	6(0.03)	0.08	1(0.05)	1(0.04)	0.51
Myocardial ischemia/infarction	26(0.13)	15(0.05)	0.65	0(0)	3(0.07)	0.28
Myocarditis	48(0.29)	5(0.02)	<0.05	5(0.31)	0(0)	0.07
Pericardial disease	22(0.13)	4(0.02)	<0.05	0(0)	0(0)	–
Supraventricular sex	71(0.39)	38(0.26)	0.18	1(0.04)	3(0.13)	0.39
Ventricular sex	3(0.01)	0(0)	0.28	0(0)	0(0)	–
Vascular AEs	702(3.83)	96(0.58)	<0.05	8(0.36)	6(0.25)	0.26
hypertension	601(3.27)	598(3.24)	0.87	5(0.19)	1(0.04)	0.18
Hypotension	59(0.36)	34(0.17)	0.31	0(0)	1(0.05)	0.57
Vasculitis	5(0.02)	5(0.02)	0.87	3(0.08)	3(0.08)	0.98
Thromboembolic events	32(0.17)	16(0.08)	0.83	4(0.13)	8(0.36)	0.14

### 3.2 Basic features

**Table 2** lists the study protocol design characteristics and the incidence rate of CAE. The current Randomized controlled trial mainly focus on the phase 3 clinical studies. The selected treatment time includes adjuvant therapy, first-line therapy, and second-line therapy. There are 16 types of tumors classified, including non-small cell lung cancer, small cell lung cancer, and urothelial cancer. There is more than one side effect in each experiment. There was a significant increase in adverse events of the Single drug ICI + Non ICI vs Non ICI trials, as well as in CAEs and vascular AEs. It may suggests that immunotherapy combined with chemotherapy may increase cardiotoxicity. Chemotherapy-related cardiotoxicity is well known, such as anthracyclines and antimetabolic drugs, the most common cardiac dysfunctions are left ventricular dysfunction, heart failure, and acute coronary syndrome. Combined with ICIs on the basis of chemotherapy may aggravate cardiac toxicity.

**Table 2:** **Research Plan Design Characteristics Table**.

-	Number	Cases Number	CAEs	Vascular AEs
Single drug ICI vs placebo	NCT0250437, NCT00636168, NCT03553836, NCT02450331, NCT03142334, NCT02125461, NCT03063450, NCT02775435,	6809	41(0.49)	15(0.18)
Single drug ICI vs Non ICI	NCT02041533, NCT02409342, NCT02220894, NCT01673867, NCT02481830, NCT01905657, NCT02580058, NCT02516241, NCT02853305, NCT03088540, NCT02555657, NCT02564263, NCT03099382, NCT02395172, NCT02576509	10257	23(0.41)	167(1.69)
Single drug ICI+Non ICI vs Non ICI	NCT03707509, NCT02576977, NCT02579863, NCT02657434, NCT02578680, NCT02367781, NCT03607539, NCT03043872, NCT02763579, NCT03434379, NCT03635567, NCT02718417, NCT02580058, NCT02853305, NCT03125902, NCT03197935, NCT03036488, NCT03141177, NCT02853331, NCT03748134, NCT03829969, NCT02494583, NCT02872116, NCT03043872, NCT02763579, NCT03066778, NCT03581786, NCT0306677, NCT02807636, NCT02425891, NCT02873195, NCT02358031	16879	124(0.57)	459(2.66)
Dual drug ICI vs Single drug ICI	NCT03158129, NCT02899299, NCT03043872, NCT02516241, NCT02872116, NCT03302234, NCT02785952, NCT02538666	4174	9(0.26)	8(0.25)

### 3.3 Deviation risk assessment

**Figure 1:**
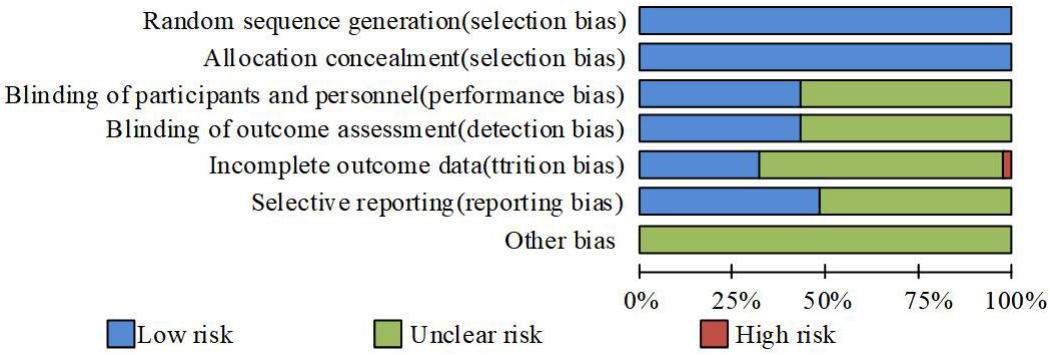
Deviation Risk Assessment.

**Figure 1** shows the risk assessment of each experiment. According to random grouping, the risk of all trials was low, mainly due to the use of publicly labeled methods. However, this does not hinder the understanding of the experiment, resulting in a certain deviation in the evaluation of the experiment. In addition, some experiments only report side effects higher than a certain value, which can have an impact on this experiment. If an experiment is terminated due to an uneven proportion of deaths between the two groups, there is a high risk of its outcome.

### 3.4 Myocarditis

**Figure 2:**
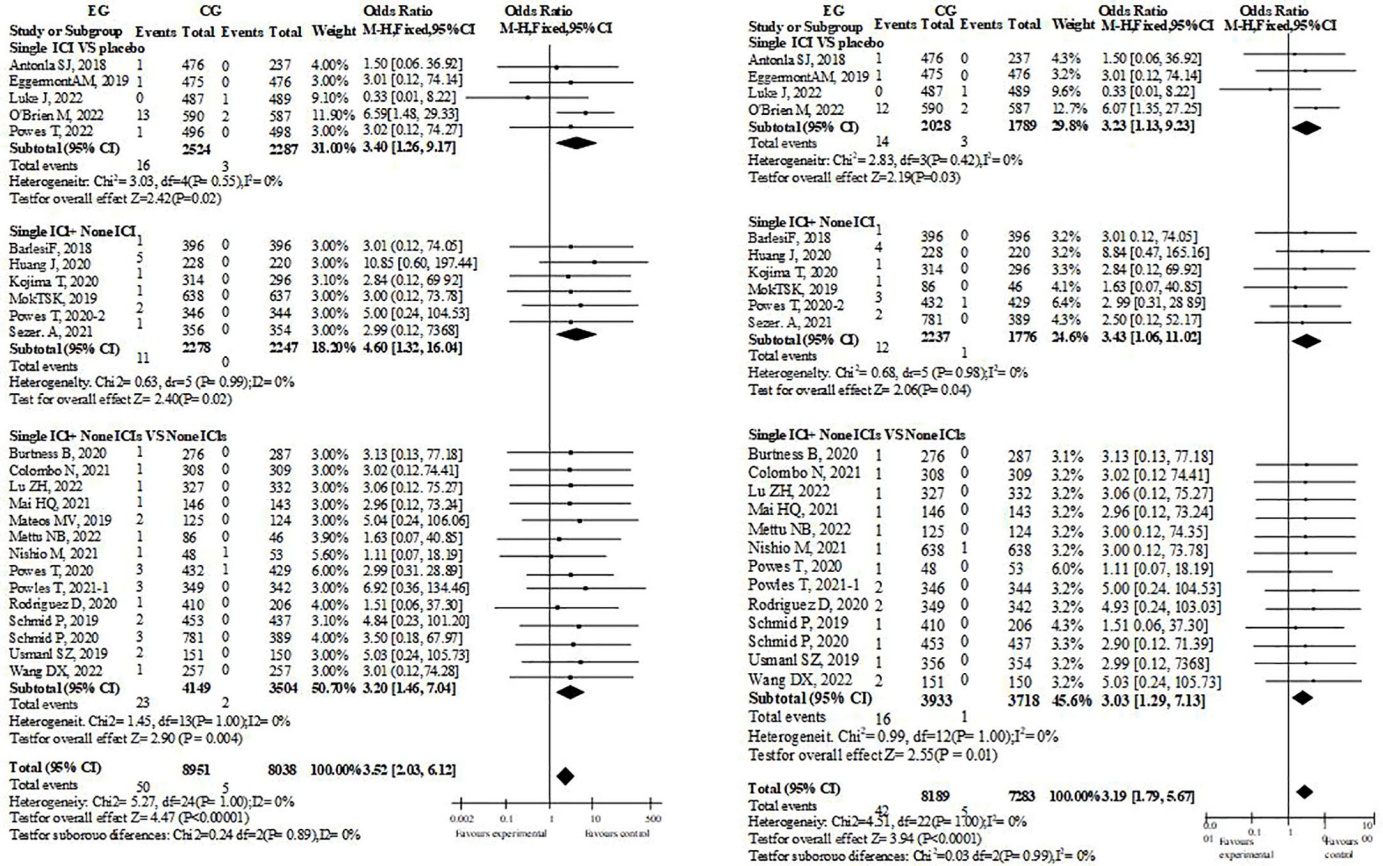
Risk assessment of AEs of Myocarditis.

There have been 29 research reports on therapeutic Myocarditis(**Figure 2**). Whether single drug ICIs are compared with placebo or not, or single drug ICIs are added to the original treatment scheme, there were significant differences in the incidence of myocarditis between EG and CG groups, and their OR values were almost greater than 1, which suggest that immunotherapy was a risk factor for myocarditis. Since ICIs are monoclonal antibodies, each study will include them in the possibility of immune related Myocarditis. However, such tendency dosen’t exist in dual drug ICIs group. Therefore, it cannot be ruled out that the credibility of dual drug ICIs is low because there are too few cases that meet the standard. This is because in the dual drug ICIs study, Myocarditis occurs in EG.

A retrospective analysis was conducted on patients with reported cardiac inflammatory diseases and compared with existing treatment methods. Pembroli ezumab is selected in most chemotherapy schemes, and the incidence rate of cardiac inflammatory disease caused by Camrelizumab is the highest., it may be related to the small number of the cases receiving Camrelizumab.At present, the reported use of Ipilimumab is a combination of two drugs, so there is a potential risk (**Table 3**).

**Table 3:** Incidence of Myocarditis in different medication regimens.

Medication	Total number of cases	Myocarditis [cases (%)]
Atezolizumab	3345	4(0.19)
Avelumab	1104	1(0.08)
Camrelizumab	351	5(1.27)
Cemiplimab	345	1(0.31)
Durvalumab	1687	3(0.18)
Ipilimumab	1896	7(0.47)
Nivolumab	4165	4(0.08)
Pembrolizumab	8052	34(0.39)
Sintilimab	938	1(0.15)
Toripalimab	2090	2(0.51)
Tremelimumab	619	0(0)
Total	22582	58(0.29)

### 3.5 Other CAEs

**Figure 3:**
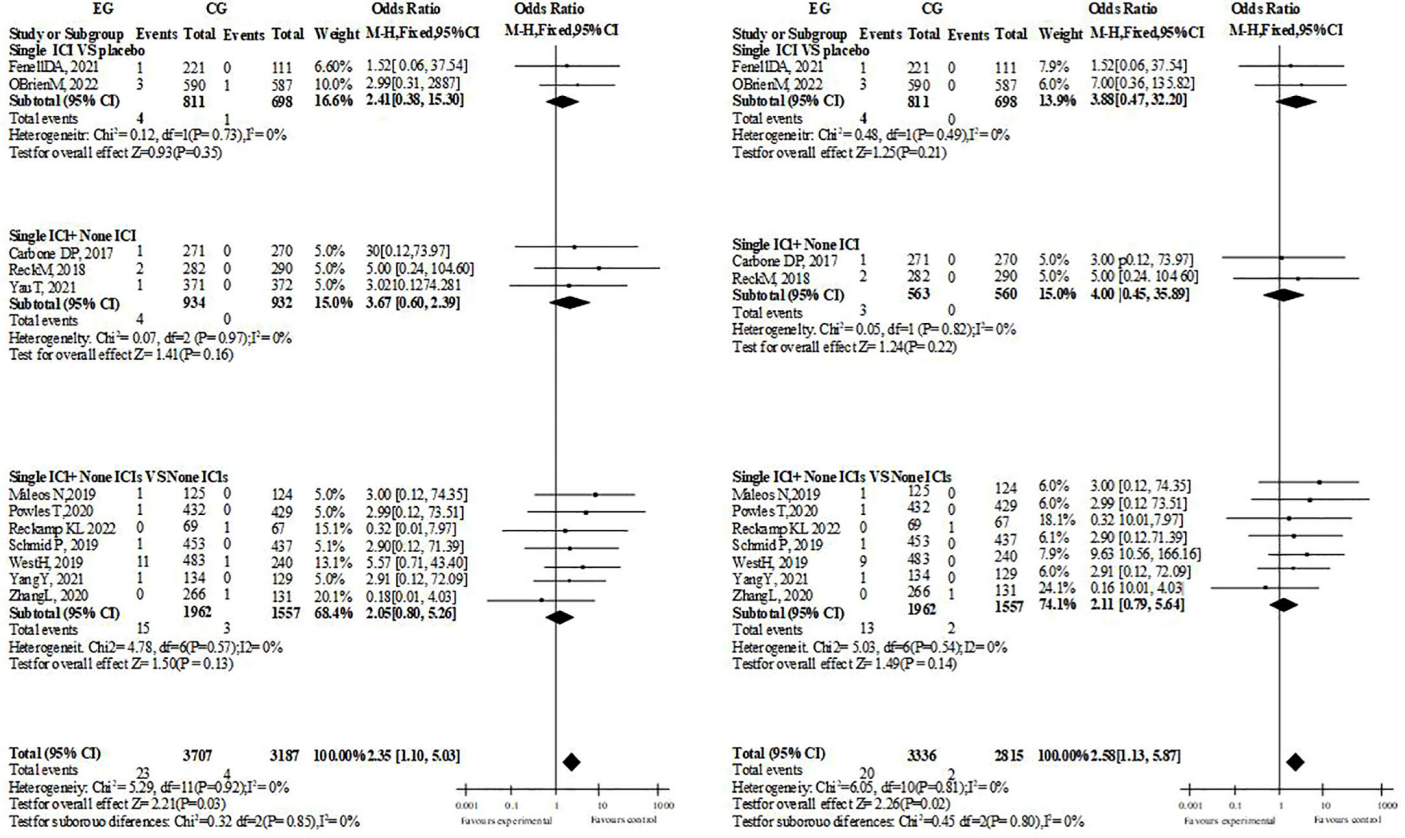
Risk assessment of AEs in pericardial diseases.

In the overall and hierarchical analysis of heart failure, cardiac arrest, and myocardial infarction/ischemia, no significant difference was found whether single drug treatment or dual drug treatment was used (3 or more side effects of ICIs treatment with single drug treatment, heart failure: OR=1.19, P=0.61. Cardiac arrest: OR=1.97; P=0.09. Myocardial ischemia/infarction: OR=1.26, P=0.43). The comparison between groups of supraventricular arrhythmias was the same (single drug treatment for ICIs with grade 3 or above: OR=0.97, P=0.90. All grades: OR=1.29, p=0.17). Among the included pericardial diseases, Pericarditis, pericardial effusion and Cardiac tamponade, the incidence of single drug ICIs was more than that of ICIs with no significant difference (OR=2.35, P=0.03, I2 = 0%, ≥ grade 3: OR=2.58, P=0.02, I2 = 0%, dual drug ICIs did not report Pericarditis, possibly because the number of included people and samples were relatively small). Although the overall necrosis rate of ICIs with a single drug is higher than that of the control, it is the same across different diseases, while the overall necrosis rate and disease are the same in ICI patients with dual drugs (**Figure 3**).

### 3.6 The incidence of related AE caused by treatment

According to **Table 4**, compared to CG, the proportion of adverse events at all levels and total serious adverse were significantly lower in patients with EG. Mortality rate was consistent between the two groups. Subgroup analysis showed that patients in EG who received ICIs+chemotherapy had significantly higher severe AEs compared to CG (P<0.05). The P-value of two groups’ death incidence is 0.14.

**Table 4:** Subgroup analysis results of incidence and risk ratio values of treatment-related AEs.

AEs	Treatment Plan	Incidence rate (95%C)		Impact assessment		Heterogeneity test	
		EG	CG	RR (95% CI)	P	P	I2(%)
Overall All Levels AEs	ICIs	0.75(0.58;0.69)	0.79 (0.83; 0.88)	0.69 (0.72; 0.77)	< 0.000	0.0005	67
	ICIs+chemotherapy	0.89 (0.95; 0.97)	0.91 (0.89; 0.96)	0.98 (1.00; 1.03)	0.04	< 0000	84
Overall severe AEs	ICIs	0.14(0.12;0.18)	0.39(0.39; 0.47)	0.35 (0.30; 0.42)	< 0.0001	< 00001	79
	ICIs+chemotherapy	0.71(0.57; 0.69)	0.61(0.45;0.59)	1.87(0.12;1.27)	< 0000	< 00001	68
Death	ICIs	0.01 (0.00;0.01)	0.03(0.01;0.02)	0.71 (0.46; 0.95)	0.02	0.85	0
	ICIschemotherapy	0.02(0.03;0.05)	0.03(0.01;0.04)	1.13 (0.93; 1.47)	0.13	0.33	0

### 3.7 Incidence rate of hematological AE

From **Table 5**, anemia and severe anemia incidence in EG is much lower than CG. Compared with CG, the neutropenia rate of EG was significantly reduced. The thrombocytopenia rate of EG was significantly lower than CG, but the incidence of severe thrombocytopenia remained unchanged. In subgroup analysis, only the incidence of severe anemia, neutropenia, and thrombocytopenia in EG with ICIs was significantly reduced (p<0.00001). The p-value between ICIs and chemotherapy in the experimental group is less than 0.05, but the p-value between two groups is greater than 0.05.

**Table 5:** Subgroup analysis results of incidence and risk ratio values of AEs in the blood system.

AEs	Treatment Plan	Incidence rate[95%CI]		Impact assessment		Heterogeneity test	
		EG	CG	RR (95% CI)	P	P	I2(%)
All levels of anemia	ICIs	0.05 (0.03; 0.07)	0.34 (0.25; 0.39)	0.15 (0.08; 0.24)	< 0.00001	0.00001	88
	ICIs+chemotherapy	0.63 (0.49;0.81)	0.62 (0.49; 0.74)	1.02 (0.96; 1.05)	0.44	0.05	42
Neutropenia of all levels	ICIs	0.01 (0.00; 0.01)	0.20 (0.18; 0.24)	0.05 (0.03; 0.09)	< 0.00001	0.07	43
	ICIs+chemotherapy	0.43 (0.32; 0.57)	0.41 (0.31; 0.54)	1.06 (1.02; 1.08)	0.02	0.46	0
Thrombocytopenia of all levels	ICIs	0.01 (0.00; 0.02)	0.12 (0.09; 0.14)	0.10 (0.08; 0.17)	< 0.00001	0.4	4
	ICIs+chemotherapy	0.33 (0.22; 0.45)	0.30 (0.21; 0.42)	1.09 (1.02; 1.17)	0.01	0.79	0
Severe anemia	ICIs	0.00 (0.00; 0.01)	0.07 (0.05; 0.12)	0.09 (0.04; 0.21)	< 0.00001	< 0.0001	73
	ICIs+chemotherapy	0.15 (0.12;0.21)	0.14 (0.11; 0.19)	1.11 (0.97; 1.28)	0.12	0.17	28
Severe neutropenia	ICIs	0.00 (0.00; 0.00)	0.14 (0.12;0.18)	0.03 (0.01; 0.04)	< 0.00001	0.87	0
	ICIs+chemotherapy	0.23 (0.17; 0.28)	0.21 (0.16; 0.28)	1.11 (1.01; 1.13)	0.02	0.67	0
Severe thrombocytopenia	ICIs	0.00 (0.00; 0.00)	0.04 (0.02; 0.08)	0.06 (0.02; 0.17)	< 0.00001	0.71	0
	ICIs+chemotherapy	0.10 (0.06; 0.15)	0.09 (0.07; 0.15)	1.16 (1.00; 1.24)	0.05	0.56	0

## 4 Discussion

As tumor immunotherapy technology rapidly develops, the efficacy of mid to late stage cancer has also significantly improved. Monoclonal antibodies (MABs) targeting CTLA-4, PD-1, and PD-L1 are currently ideal drugs for the treatment of solid and vascular tumors. The antibodies represented by Ipilimumab, with CTLA-4 as the core, have greatly improved the efficacy of this type of tumor (5, 6). Subsequently, monoclonal antibodies targeting PD-1 and PD-L1 emerged one after another and began to be applied in the clinical treatment of various cancers. When the effect of such anti tumor drugs is definite, it may also cause a variety of side effects related to body immunity, such as Myocarditis. Myocarditis has been observed to be more frequently present in combination therapy and with anti-PD-1/anti-CTLA-4 therapy. The incidence of myocarditis varies between different classes of ICIs, with anti-PD-1 agents having the lowest incidence (0.5%) and anti-CTLA-4 monotherapy having the highest (3.3%). However, it is important to note that these differences may be overestimated and that both anti-PD-1 and anti-PD-L1 agents have a similar toxicity profile (7).It has been reported that PD-1 and PD-L1 have high levels of expression in heart tissue, and PD-1 and CTLA-4 can cause Myocarditis (8, 9). In recent years, several cases of central myocarditis and fatal cardiac dysfunction have been reported in tumor patients due to ICIs. The mechanism of cardiotoxicity is still under investigation, and the main reason may be that PD-L1 expressed in human myocardium is involved in protection against immune-mediated cardiac injury and inflammation (10).

A multicenter retrospective study reported cardiac fibrosis in a melanoma patient treated with Ipilimumab. In addition, there are also literature reports on patients who develop CAE after using iprivumab in clinical practice. Multiple studies have explored the cardiotoxicity related to the body’s immune response and its relationship with disease outcomes. In a multicenter study, six patients had poor prognosis, with two patients experiencing cardiotoxic effects under the action of Ipilimumab. Among these six patients, despite receiving appropriate treatment, two still died. In recent years, studies have reported two cases of acute Myocarditis caused by ICIs (11, 12). Two patients died after taking a checkpoint inhibitor once, even after undergoing intensive treatment. Both patients had hypertension, but there were no other cardiovascular risk factor. Pathological section showed a large number of lymphocytes and macrophages infiltrating in myocardium, sinus and Atrioventricular node. PD-L1 is significantly upregulated in ischemic heart tissue and tissues. Researchers conducted research on T lymphocytes infiltrating the heart, skeletal muscles, and cancer, and the results confirmed that TCR is replicated. There are many risk factors related to cardiovascular toxicity reactions related to ICIs, and analyzing these risk factors can better guide the condition of cancer patients and monitor their cardiac indicators (13, 14). The first is the risk factor associated with ICIs. Compared with the single drug, the incidence of Myocarditis associated with dual drugs is higher, and the age of Myocarditis is also earlier. A retrospective study showed that among 30 patients with cardiac toxic side effects related to ICI, 23 patients with Myocarditis were male, with an average age of 72 years. In addition, many registered clinical trials in the past found that more than 60% of patients with Myocarditis related to ICI were men. In addition, combined use with drugs such as anthracycline can also increase the risk of myocardial virus infection in ICI patients. In fact, many tumor patients have received radiation, chemical or tumor Targeted therapy before the emergence of ICIs (15, 16). This therapy can allow antibodies in myocardial tissue to come into contact with myocardial tissue and generate a specific immune response in the tissue. Inhibitors can affect the blood flow in the heart and coronary arteries through two different receptors of platelet origin, thereby increasing cardiovascular disease risk. Other possible risk factors include the presence of cardiovascular disease or previous left ventricular insufficiency related to radiotherapy or chemotherapy.

This study carried out a meta-analysis of RCT. CAE risk in patients with ICIs was higher than that in patients without ICIs, especially the incidence rate of Myocarditis was significantly higher. Due to the coverage of a large number of patients, various types of cancer, and strict RCT, the mechanism of myocardial injury in ICIs will be further clarified. ICIs associated Myocarditis has unique characteristics, and patients often have serious side effects above grade 3, which seriously threaten the survival of patients. And the lack of effective clinical data poses great challenges to the study of its pathogenesis. After the inhibition or gene silencing of molecules such as PD-L1/2 and CT-LA-4, the cardiac inflammatory response significantly increases, accompanied by inflammatory reactions. Because Myocarditis related to ICIs may not have special clinical signs, complete auxiliary detection is particularly important. Meta analysis shows that on the electrocardiogram, each type will show differences. More commonly, in these cases, the most common one is a signal transmission disorder, and for this situation, serological detection methods are relatively sensitive, but have no specificity. Myocardial biopsy is the best examination method. Although it serves as the ‘gold standard’, it is not suitable to adopt it due to its high risk (17, 18). For suspected ICIs related Myocarditis, the first choice is hormone stimulation, and the second choice is commonly used Immunosuppressive drug. Therefore, under the anticancer effect of ICIs, how to enhance their protective effect on the heart is an urgent issue that requires in-depth research. The work systematically analyzed the Myocarditis events that may be related to the immune factors of the body. There is no difference between Myocarditis and pericardial lesions, but the possible risk factors should not be ignored.

Myocarditis is the most common cardiovascular disease in clinic, with clinical manifestations of dyspnea, syncope or Hypotension, accompanied by obvious arrhythmia (19). When angina pectoris is related to ICI, it may be manifested as increased Troponin or abnormal electrocardiogram (ECG). Acute congestive heart failure is the main clinical symptom, accompanied by a decrease in left ventricular contractility, but nearly half of heart failure patients are accompanied by a severe decrease in cardiac contractility (20). The main clinical manifestations of myocarditis caused by ICI are new segmental and diffuse contraction of left ventricle, and obvious inflammatory reaction. The second common cardiac complication of ICIs is Pericarditis. It is characterized by isolated pericardial effusion and Cardiac tamponade. From the data of World Health Organization, the average time between ICI of more than 3000 cases related pericarditis and the occurrence of cardiac toxic events is 30 days (21). Men are more prone to heart toxicity than women. In most cases of pericarditis, there are special electrocardiogram changes. Echocardiography and magnetic resonance imaging are of great value in oericarditis diagnosing. Due to the lack of specific research on its potential mechanism and animal models of HD, the oathophysiology of ICI has not been fully understood. However, in the limited human postmortem histology, a large number of lymphocytes may be seen infiltrating into the epicardium, which indicates that the immune induced inflammatory response mediated by T cells may be crucial to pericarditis development. The diagnostic criteria for ICI related pericardiocentesis are not unified (22, 23). The diagnosis of Pericarditis may be on the foundation of one following condition at least: acute pleural and chest pain, pericardial friction during surgery, ST segment elevation of ECG and pericardial effusion on echocardiography. Different types of arrhythmia are also the main characteristics of cardiac toxicity related to ICI. Atrial fibrillation, ventricular arrhythmia and conduction disorder are the most common. Conduction system diseases caused by ICI are considered a serious and fatal heart disease. One possible mechanism may include inflammation of the T cell infiltration conduction system (24, 25). ECG indicates a long PR, class II heart block, and the pacemaker placement needs to be properly relaxed, because the heart block in patients with acute Myocarditis is usually very fast. In addition, when using ICIs without evidence of Myocarditis, conduction disorder may be a rare and potentially fatal result. To prevent death, the study suggests conducting regular electrocardiogram tests on all ICIs. After detection of cardiac conduction block or bradycardia, more thorough examinations should be carried out, such as Holter monitor and echocardiography (26, 27). Cardiac MRI can provide more information about the state of cardiac injury and subclinical inflammation. To evaluate the time of discontinuation and implementation of ICI, cardiac surgeon intervention is very important. Cardiac complications can lead to serious consequences and even death, while interruption of cancer treatment increases the risk of disease progression and whether subsequent treatment can be applied again with ICIs. In one case report, a 55-year-old female patient diagnosed with metastatic malignant melanoma was treated with an immunotherapy combination of Ipilimumab and Nivolumab. However, the patient developed grade 3 myocarditis and then stopped immunotherapy and began corticosteroid therapy until full recovery. Soon after the patient developed tumor metastasis, Nivolumab monotherapy was restarted to minimize the risk of further adverse reactions. The patient has received 41 cycles of treatment with no adverse events and achieved complete remission (28). In addition, a retrospective “real world” database study published in 2020 concluded that retreatment with anti-PD-1 monotherapy may provide additional benefits for patients with advanced melanoma who responded to the first course of anti-PD-1 monotherapy with CR, PR, or SD (29). While alternative treatment options should be initiated based on the patient’s history of immune-related adverse events and NCCN guidelines (which recommend permanent discontinuation of immunotherapy in such cases), management of immune-related adverse events should be individualized and guided by the patient’s clinical presentation and cardiac function. The meta-analysis of this study has certain limitations. Firstly, when comparing ICIs with normal CG, there are differences in the efficacy of ICIs, and most studies have not considered the pathogenesis of ICIs. Secondly, the study did not use the Meta method to analyze patient data, making it impossible to evaluate other key clinical factors related to adverse cardiovascular reactions, such as gender, age, tumor type, number of treatment lines, etc. In a search of the U.S. Food and Drug Administration Adverse Event Reporting System (FAERS) database, ICI related cardiovascular toxicity appeared to affect more men (56.90%) than women (36.79%). The median age was 67 years (interquartile [IQR] 58–74 years). ICI related adverse cardiovascular events were most common in patients with lung cancer, pleural cancer, thymus cancer and heart cancer (34.49%) (30). Thirdly, this study only included RCT, which can compare ICI with non ICI, dual drug ICI with single drug ICI. Despite existing statistics, it is still not possible to comprehensively evaluate the CAE risk of these patients, and some studies only report a specific CAE risk (31, 32). In RCT, due to the strict requirements of the trial protocol, the trial needs to be discontinued in the event of severe cardiotoxicity, so very fewer cases can be collected to rechallenge of ICIs after the incidence of cardiac toxicity. Meta results showed that patients with ICIs were more likely to have cardiovascular side effects than those without ICIs, especially ICIs related Myocarditis and pericardial lesions. The implementation of this project will lay a solid theoretical foundation for the use and safety evaluation of ICIs. For patients treated with ICIs, we need comprehensive monitoring and multiple follow-up visits so that we can ensure the efficacy of treatment and promptly detect and intervene in any adverse events that may arise.

## Data availability statement

The raw data supporting the conclusions of this article will be made available by the authors, without undue reservation.

## Author contributions

XL: Writing – original draft, Writing – review & editing. DL: Writing – review & editing.
